# Assortative marriages by body mass index have increased simultaneously with the obesity epidemic

**DOI:** 10.3389/fgene.2012.00125

**Published:** 2012-07-18

**Authors:** Teresa A. Ajslev, Lars Ängquist, Karri Silventoinen, Michael Gamborg, David B. Allison, Jennifer L. Baker, Thorkild I. A. Sørensen

**Affiliations:** ^1^Institute of Preventive Medicine, Copenhagen University HospitalCopenhagen, Denmark; ^2^Population Research Unit, Department of Social Research, University of HelsinkiHelsinki, Finland; ^3^Nutrition Obesity Research Center, University of Alabama at BirminghamAL, USA

**Keywords:** assortative mating, body mass index, childhood, obesity, overweight, phenotype, human genetics

## Abstract

**Background:** The genetic predisposition to obesity may have contributed to the obesity epidemic through assortative mating. We investigated whether spouses were positively assorted by body mass index (BMI; = kg/m^2^) in late childhood, and whether changes in assorted marriage by upper BMI-percentiles occurred during the obesity epidemic. **Methods:** In the Copenhagen School Health Records Register (CSHRR) boys and girls with measures of BMI at age 13 years later became 37,792 spousal-pairs who married between 1945 and 2010. Trends in the spousal BMI correlations using sex-, age-, and birth cohort-specific BMI *z*-scores across time were investigated. Odds ratios (ORs) of marriage among spouses both with BMI *z*-scores >90th or >95th percentile compared with marriage among spouses ≤90th percentile were analyzed for marriages entered during the years prior to (1945–1970), and during the obesity epidemic (1971–2010). **Findings:** Spousal BMI correlations were around 0.05 and stayed similar across time. ORs of marriage among spouses with BMIs >90th percentile at age 13 were 1.21, 1.05–1.39, in 1945–1970, and increased to 1.63, 1.40–1.91, in 1971–2010 (*p* = 0.006). ORs of marriage among spouses both >95th BMI percentile were higher and increased more; from 1.39, 1.10–1.81, to 2.39, 1.85–3.09 (*p* = 0.004). **Interpretation:** Spousal correlations by pre-marital BMIs were small and stable during the past 65 years. Yet, there were assorted marriages between spouses with high BMI at age 13 years and the tendency increased alongside with the obesity epidemic which may increase the offsprings' predisposition to obesity.

## Introduction

The obesity epidemic has developed so rapidly that corresponding changes in environmental influences must have played a major role (McAllister et al., 2009). However, changes in the genetic influence on obesity may also have contributed (Rokholm et al., 2011). If positive assortative mating by body mass index (BMI; = kg/m^2^) exists, as suggested by several studies, (Allison et al., 1996; Hebebrand et al., 2000; Katzmarzyk et al., 2002; Silventoinen et al., 2003; Jacobson et al., 2007; Speakman et al., 2007; Di Castelnuovo et al., 2009) it may assemble obesity-promoting risk alleles and possibly also environmental influences that interact with the genes in families (Hebebrand et al., 2000; Katzmarzyk et al., 2002; Jacobson et al., 2007; Li et al., 2009; Power et al., 2011).

Furthermore, if positive assortative marriages by pre-marital BMI have increased, as we hypothesize, they may, at the population level, have amplified the obesity epidemic, which in Denmark is concentrated in the upper end of the BMI distribution (Thomsen et al., 1999). Additionally, the obesity epidemic in Denmark developed in phases, alternating between periods of stability and periods of an increasing prevalence of obesity. Birth cohorts from the year 1942 to the early 1950s and from the year 1971 and onwards have shown steeply increasing prevalence of obesity that is already present at school ages, whereas the prevalence was stable in the years before 1942 and from the early 1950s until the take-off after 1970 (Olsen et al., 2006). In the recent decade the obesity prevalence has again stabilized (Pearson et al., 2010; Rokholm et al., 2010). The aim of the present study was to examine if positive assortative marriage by phenotypic similarities in BMI before the spouses met occurred, and whether such spousal resemblance in BMI increased during the development of the obesity epidemic by aligning the time of marriage with the shifting nature of the Danish obesity epidemic.

## Materials and methods

**Figure 1:**
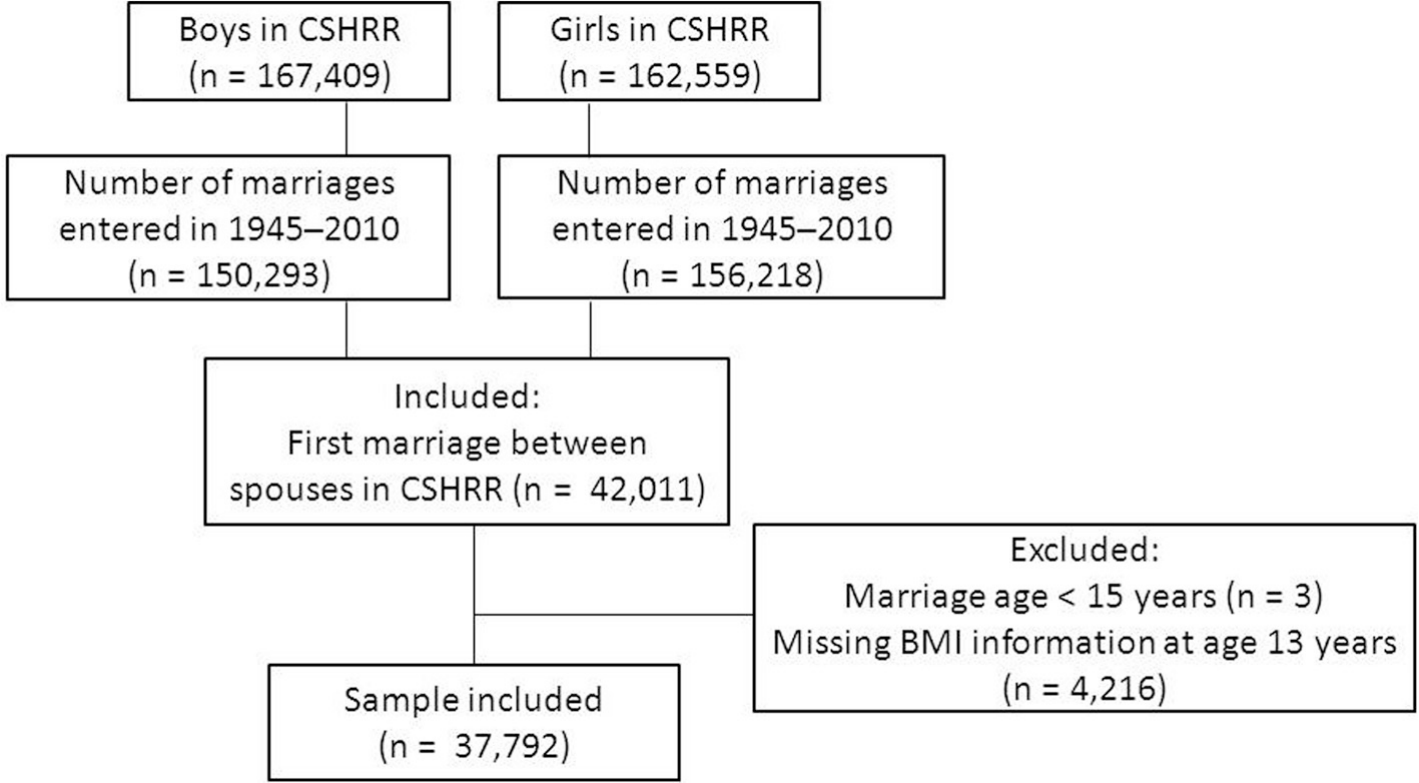
**Flowchart of the study population**.

The source population for the study was The Copenhagen School Health Records Register (CSHRR), which contains information on 372,636 children, born during the years 1930–1989, who underwent health examinations in public and private schools in the Copenhagen municipality (Baker et al., 2009). The records include measurements of height and weight routinely performed by health doctors and nurses. The present study included 167,409 boys and 162,559 girls for whom the unique person identification number, assigned to all Danish citizen alive from 1968 and onwards, was available. Using the person identification numbers, the participants were linked via a national register on vital statistics in Statistics Denmark to the marriage register. This procedure led to the identification of 306,511 marriages during the period from 1945–2010 of either a boy or a girl in the CSHRR. Of these marriages, 42,011 were the first marriage among spouses who both were included in the CSHRR (Figure 1). Three pairs were excluded due to recorded marriage age under 15 years. Information on height and weight at age 13 years was available in the CSHRR for 37,792 pairs, which formed the final study sample.

The Danish Data Protection Agency has approved the use of the CSHRR for this study (according to Danish law, ethical approval is not required for purely register-based studies).

### Measures of body mass index at age 13 years

We calculated each child's BMI from heights and weights measured between ages 12 and 14 years. Sex- and age- specific BMI *z*-scores were generated. All children measured from years 1955–1960 constituted the internal reference population. Data from these years were used because the prevalence of overweight was low and stable during this period. BMI *z*-scores were calculated by subtracting BMI for each child from mean BMI in the reference population, divided with the standard deviation in the reference population. The *z*-scores were interpolated to exact ages if two surrounding measurements were available or extrapolated to exact ages if only one measurement was available (Baker et al., 2007). These values were then standardized^1^ according to gender and nine birth-year intervals (1930–1934, 1935–1939, 1940–1944, 1945–1949, 1950–1954, 1955–1959, 1960–1964, 1965–1969, and 1970–1989; last interval was extended owing to a decline in the size of the married population). The included study populations BMI distribution was validated against the total CSHRR-cohort as well as against the total ever-married population in the CSHRR (excluding the study population) by two intervals of birth year.

### Marriage by BMI percentile groups

Boys and girls were grouped into categories according to their gender- and birth cohort- specific BMI *z*-scores; ≤25th, >25th–75th, >75th–90th, >90th–95th, and >95th percentile. The narrower categories in the upper part of the BMI-distribution, >90th–95th, and >95th percentiles, corresponds approximately to international criterias of overweight and obesity, respectively (Cole et al., 2000).

### Secular trends

Two periods of marriage year (1945–1970, and 1971–2010) were chosen to investigate secular changes in assortative marriages occurring concomitantly with the development of the obesity epidemic, which in this sample evolved by a steep rise among individuals born after 1970 following a stable level the years before (Olsen et al., 2006). Supplemental analyses were conducted using four categories of marriage year (1945–1960, 1961–1970, 1971–1980, and 1981–2010) to see if the observed trend was monotonic or irregular.

### Statistical analyses

The BMI distributions were summarized according to gender and the chosen two marriage periods by the five percentile cut-offs; 25th, 50th, 75th, 90th, and 95th, respectively. The birth cohort standardized BMI-*z* scores of boys and girls were used in spousal-correlation analyses by 10 marriage-year intervals as well as by two marriage periods using linear regression (BMI-based variables standardized in order to make regression- and correlation analyses equivalent). Differences in estimates over the two and 10 marriage periods were respectively tested by including an indicator for marriage period and a categorical time variable for marriage year (time since first period; each period represented by the midpoint of the corresponding interval), as well as corresponding interaction terms with the girls standardized BMI *z*-scores, in the model. Ratios of observed marriage frequencies to expected frequencies under the assumption of random marriage were estimated, and deviations from random marriage were assessed by Chi-square tests. Logistic regression analyses were used to investigate changes between the two marriage periods in the OR's of boys with BMI *z*-scores above the 90th or 95th percentiles marrying girls similarly above the 90th or 95th percentiles. In both analyses, the reference population of the dichotomized BMI groups of boys and girls, were those below and equal to the 90th BMI *z*-score percentile. The differences across marriage periods were tested by including an interaction term in the model between the dichotomized BMI of girls and marriage period. Height and marriage age were considered as possible confounders, and marriage ages, as well as gender- and age-specific *z*-scores of heights at age 13 years, were included jointly as continuous adjustment variables in the logistic regression models. The prevalence of pairs in which both spouses were above the 90th or 95th BMI *z*-score percentile were calculated and compared to the expected prevalence under random marriage being (1–0.90) × (1–0.90) × 100 ~ 1 percent, and (1–0.95) × (1–0.95) × 100 ~ 0.25 percent, respectively. All analyses were conducted in Stata 11.2, StataCorp 2009 LD, Texas, USA.

### Role of the funding source

The funders had no role in the study design, analyses, or preparation of the manuscript.

## Results

### BMI distributions according to marriage year

The median BMIs in boys and girls at age 13 years were stable across the two marriage periods from 1945 to 2010 (Table 1). Whereas the lower BMI percentiles were virtually unchanged, the upper percentiles (90th and 95th) increased in both boys and girls across the two periods, reflecting the obesity epidemic (Table 1). The age at marriage also increased from mean of 23.8 years SD (3.3) and 21.6 years (3.1), respectively, in boys and girls, in the years of 1945–1970, to 29.7 years (7.5) and 27.9 years (7.6) in the years of 1971–2010. Likewise, mean heights increased slightly from 152 cm SD (7.4) to 154 cm (7.8) in boys at age 13 years, and from 154 cm (6.9) to 156 cm (7.1) in girls, respectively. The BMI distributions of our study sample stratified by gender and two birth cohorts were similar to the distributions in the total- as well as in the ever married CSHRR-population. Yet, the 95th BMI percentile in our married girls born in years 1971–1989 was 24.9 kg/m^2^ at age 13 years, whereas in both the total CSHRR- as well as in the ever married CSHRR-population (without the study population) it was 25.9 kg/m^2^.

**Table 1:** **BMI distributions according to two periods of marriage year—before and during the obesity epidemic**.

**Marriage year**	**Boys BMI at age 13 years**							**Girls BMI at age 13 years**						
	**Mean**	**SD**	**Percentile**					**Mean**	**SD**	**Percentile**				
			**25**	**50**	**75**	**90**	**95**			**25**	**50**	**75**	**90**	**95**
1945–1970 (*n* = 21, 984)	18.1	1.9	16.8	17.8	19.1	20.5	21.5	18.5	2.2	17.0	18.3	19.8	21.3	22.5
1971–2010 (*n* = 15, 808)	18.1	2.1	16.6	17.8	19.2	20.9	22.1	18.5	2.4	16.9	18.2	19.8	21.7	23.0

### Spousal BMI correlations

**Figure 2:**
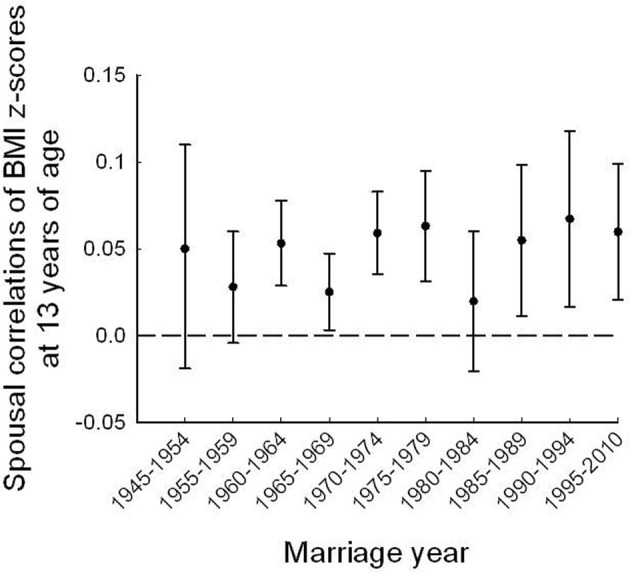
**Spousal correlations for standardized BMI *z*-scores from age 13 years with 95% CI estimates, by 5-year intervals of marriage cohort**.

Spousal correlations of BMI *z*-scores at age 13 years by intervals of marriage-years were stable over the 10 marriage periods (Figure 2), with no significant trend over time (*p* = 0.25). Similarly, estimated correlations using only two marriage periods (before and during the obesity epidemic) showed no significant difference either (*r* = 0.04, 95% CI 0.02–0.05, and *r* = 0.06, 95% CI 0.04–0.07, respectively (*p* = 0.11). Adjustments for marriage age and height at age 13 years did not attenuate the coefficients (*r* = 0.05, 95% CI 0.03–0.05, and *r* = 0.06, 95% CI 0.05–0.08, respectively).

### Observed versus expected ratios of marriage by BMI

**Figure 3:**
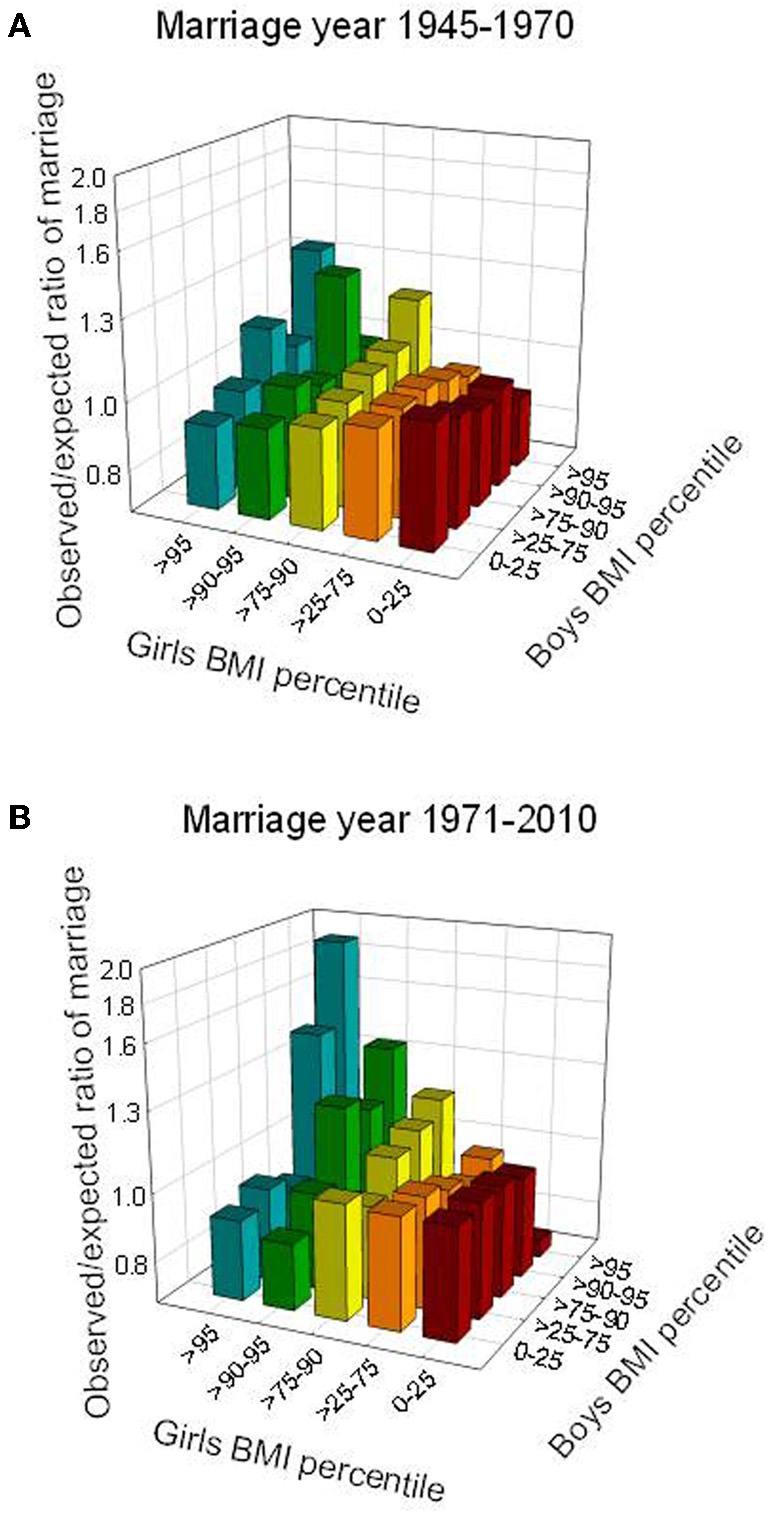
**Observed compared to expected ratios of marriage (log scale) according to group-based BMI *z*-scores at age 13 years, shown for two marriage periods, prior to (A) and during (B) the obesity epidemic.** Ratio = 1 corresponds to random marriage. Pearson's Chi squared test, *p*-values; *p* = 0.005 (1945–1970) and *p* < 0.0001 (1971–1989).

Across the BMI *z*-score percentile groups of spouses at age 13 years, marital frequencies were around one among spouses who both were below the 90th percentile (Figure 3). In contrast, striking non-random upward deviations were found for spousal pairs in which both were above the 90th BMI *z*-score percentile at age 13 years, and the deviations increased in the last marriage-period (after 1970) as compared with the earlier period. In the group where both spouses were above the 95th percentile at age 13, the marriage ratio was clearly stronger—1.89 vs. 1.34 times the expected frequency—in the last compared to the first marriage period (Figure 3).

### Assortment by upper percentile BMI

**Figure 4:**
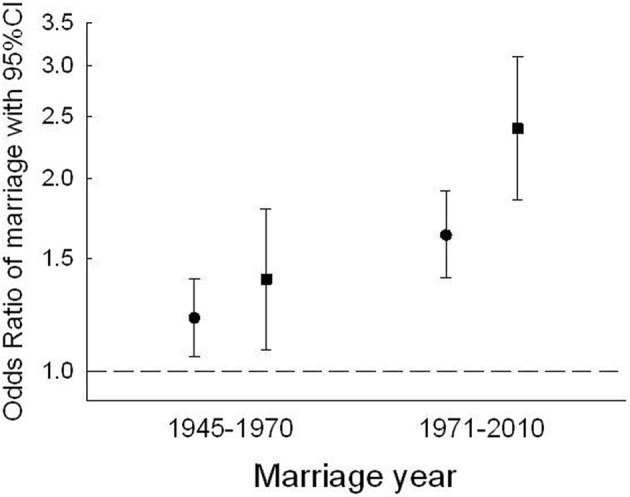
**Odds Ratios of boys with BMIs above the 90th (●) or 95th (■) percentile marrying girls similarly above the 90th or 95th BMI percentile at age 13 years compared to marriages among pairs under or equal to the 90th percentile.** Differences by marriage periods *p* = 0.006, and *p* = 0.005, in pairs above the 90th and 95th BMI percentiles, respectively. ORs are adjusted for height at age 13 years as well as marriage age.

The OR of a boy above the 90th BMI *z*-score percentile at age 13 years marrying a girl similarly above the 90th percentile at age 13 was 1.21, 95% CI 1.05–1.39 in the marriage years from 1945–1970, and increased to 1.54, 1.33–1.79 in marriage years from 1971 to 2010. This corresponds to an increase in prevalence from 1.14 to 1.45 percent between the two marriage periods (*p* = 0.006), to be compared with the random marriage frequency being 1.0 percent in this group. For spouses both above the 95th BMI *z*-score percentile at age 13 years, the OR of marriage compared with marrying spouses who were below the 90th BMI percentile at age 13 years, also increased, from OR 1.41, 1.01–1.81, in the early marriage period to 2.22, 1.74–2.82, in the last marriage period, respectively. Compared with an expected random marriage frequency of 0.25 percent in this group, the observed prevalence of spousal pairs was higher and increased from 0.32 to 0.48 percent (*p* = 0.024) by the two marriage periods. The ORs of concordant marriages by high BMI were strengthened by adjustments for height at age 13 years and marriage ages. Estimates increased from 1.21, 95% CI 1.05–1.39 in 1945–1970 to 1.63, 1.40–1.91 in 1971–2010 in pairs both above the 90th percentile (test for difference, *p* = 0.006), and even more strikingly from 1.39, 1.08–1.79 to 2.39, 1.82–3.09 in pairs both above the 95th percentile (test for difference, *p* = 0.005) (Figure 4). Supplemental analyses using four marriage periods confirmed the changes in marriage patterns by high BMI starting around year 1970 (not shown).

## Discussion

This study showed small, and stable spousal correlations around 0.05 of measured, and gender- and period-specific standardized BMI values at age 13 years in the period from 1945 to 2010. However, analyses using the distributions of spousal BMIs at age 13 years revealed striking heterogeneity in assortment, with a possible threshold around the 90th BMI percentile. In the upper end of the BMI distribution we observed significantly increased frequencies of assorted marriages between those who both were above the 90th BMI *z*-score percentile at age 13 years and even more so among pairs both above the 95th percentile.

While the upper percentiles moved upwards during the development of the obesity epidemic, we observed that the tendency for positively assorted marriage above the upper percentiles increased exactly around the year of the second take-off of the obesity epidemic in this sample. In addition, previous studies have shown that in Denmark as well as in other parts of the world, (Toschke et al., 2008; Beyerlein et al., 2010) it is not the majority of the Danish children who have become heavier, rather it is a tail phenomenon in the upper end of the BMI distribution. The pattern of assorted marriages was not affected by marriage ages or heights even though both increased during the time period under study.

This study is based on childhood BMIs measured by school doctors or nurses at the health examinations in the schools of the central Copenhagen municipality. The CSHRR contains a unique and prospectively collected set of measurements of height and weight on virtually all children in these schools. The cohort represents all social classes, and thus selection or measurement bias in terms of who has or has not been measured is minimal (Baker et al., 2009).

Although societal changes occurred in this area during the period of the study, we have no reason to suspect that the results of the study are dependent on these changes as suggested by the stability of the overall spousal correlations. The national registers allowed us to identify marriages irrespective of the spouses' areas of residence at any age. Marriages between spouses who both had attended schools in the Copenhagen municipality encompassed 20.3 percent of the original CSHRR-population. There were no indications of BMI differences between our study sample and the total- or ever-married CSHRR-population. However, compared with these groups, the BMI value at the 95th percentile was slightly lower among girls in our married cohort. Comparing our study population with the total ever-married population in the CSHRR (not including our study population) as well as with the total CSHRR, we did not observe differences in marriage age or median BMIs within the five BMI groups (not shown). These findings suggest that the women with high BMIs at age 13 years do get married but that they tend to marry a spouse who did not attend school in the Copenhagen area. Supplemental analyses showed that the marriage frequencies within the CSHRR were lower among those above the upper percentiles. Thus, those who married within the CSHRR population and who had a BMI above the 95th percentile were 72% of the 5% expected. Those who married and had a BMI between the 90th and the 95th percentile were 91% of the 5% expected. Still, as suggested by the BMI estimates from the total ever-married population they may have married spouses outside the CSHRR-population. Also, there were no time trends across the two marriage periods in these frequencies. In general, rates of marriage decreased from 1965 to 1980 in Denmark. As this trend overlaps with both marriage periods, it should not have biased our findings.

Previous studies have reported correlations in spousal BMI values ranging from 0.10 to 0.15 (Allison et al., 1996; Katzmarzyk et al., 2002; Silventoinen et al., 2003; Jacobson et al., 2007; Speakman et al., 2007; Di Castelnuovo et al., 2009). Only one of these studies, however, used premarital BMIs, (Allison et al., 1996) and thus the results of a correlation at 0.13 were presumably not influenced by cohabitation. Assortative mating by high BMI has also been studied previously, (Hebebrand et al., 2000; Jacobson et al., 2007), however, with the limitation of only having current or recalled weights available. In a clinical series of very obese children, it was found that the majority of the children's parents, -both had an age-specific BMI among the highest 10 percent of the German population according to a national reference, even when recalled weights from age 20 were used (Hebebrand et al., 2000).

Our study, using measured heights and weights from age 13 years, avoids biases from recalled weights as well as the possible influence of cohabitation on BMI in adult life before and after the marriage. As such, the overall spousal correlations in our study are expected to be lower than those observed around the time of marriage. Changes in BMI between age 13 years and the age at marriage occur, making the tracking correlations approximately at 0.7 between late childhood and adulthood, (Herman et al., 2009; Silventoinen and Kaprio, 2009) may explain the lower spousal correlations found in the present study. The differences between the spousal correlations by BMI at age 13 years and at the age of marriage suggest that our findings of assortative marriages at the upper end of the BMI distributions are conservative estimates. The ages of marriage increased over the two time periods and may thus have made the increase in assortment at the higher BMI levels over time even more pronounced than found here. However, the increase in marriage ages were not dependent on the BMI levels, neither at the higher levels, and adjustments for marriage ages in the present study and in previous studies did not alter the spousal correlations (Allison et al., 1996; Speakman et al., 2007). Some children in the CSHRR were missing data on height and/or weight at the ages of 12–14 years, and thus were not eligible for inclusion in this study as their BMI at 13 years could not be calculated. The most likely explanation for the missing data is that the family moved out of the Copenhagen school district, and thus the children were not measured in the Copenhagen school district after they moved. To examine if there was a pattern in the missing data, we compared the BMI values at seven years of age among those included in our study who were missing a BMI value at age 13 with those in the entire cohort. We found very similar BMI values at seven years of age in these two groups, and there was no time trend in the missing data.

Assortative mating by height has been described previously (Silventoinen et al., 2003). In our sample, the correlations between height and BMI *z*-scores across the time intervals were approximately 0.30 and the height of the children increased over the time period studied. However, adjustments for height at age 13 years did not alter the spousal correlations; neither did it change the ORs of marriage by high BMI values.

Genetic predisposition toward a high BMI in both spouses—apparent already in childhood—may predispose their offspring towards the same trait to a higher degree than by simply adding the effects (Crow and Felsenstein, 1968). The offspring may inherent several interacting risk alleles from both parents, and, in addition, homozygosity at loci associated with obesity may allow recessive loci to be expressed (Allison et al., 1996; Hebebrand et al., 2000; Katzmarzyk et al., 2002; Silventoinen et al., 2003; Jacobson et al., 2007; Speakman et al., 2007). Epigenetic markers may be transferred between generations, possibly influencing offspring gene-expression (Plomin, 1990; Morgan and Whitelaw, 2008, 2009). In addition, obese spouses may increase the influence from the obesogenic environment, for example through social factors, (Fontaine et al., 2011) that correlates with and interacts with the genes. A recent longitudinal twin study suggested that different gene sets influence the tracking level of BMI and the rate of change in BMI between adolescence and young adulthood (Silventoinen and Kaprio, 2009). Thus, the effects of assortative mating observed by high parental BMIs in adolescence may influence offspring BMI levels in adolescence, independently of the parents BMI in adulthood. Although our cohort does not hold genetic data, our findings strongly encourages further investigations of the molecular genetic implications of upper BMI assorted parents for the occurrence of obesity in subsequent generations.

Assortment by upper BMIs increased exactly around the birth year where the second increase in prevalence of obesity among children was observed. With available offspring BMI data on a subpopulation in this cohort, we are currently investigating changes in the intergenerational transmission. Whether assortative marriages by high BMI have contributed to the obesity prevalence on the population level depends on a number of factors such as offspring risk conditioned on parental BMI-combination, (Jacobson et al., 2007) and the number of offspring in each family, which again is dependent on fecundity, (Clark and Spuhler, 1959) and divorce rates (Allison et al., 1996).

Assortative marriages by high BMI values, and the particular observed increase in assortment especially in pairs with very high BMI values already at age 13 years may be due to not only BMI as such, but different psychological, social as well as bodily factors interacting and correlated with BMI (Wardle et al., 1995; Allison et al., 1996; Katzmarzyk et al., 2000; Speakman et al., 2007; Di Castelnuovo et al., 2009; Sear and Marlowe, 2009; Danielsdottir et al., 2010). This area deserves detailed investigations in order to identify causes and possible unwanted and adverse effects in subsequent generations, with the simultaneous aim of identifying possible targets and tools for prevention, while also considering the ethical aspects.

## Conclusion

General correlations in pre-marital BMI values between spouses were small and stable during the past 65 years. Having a high BMI in late childhood, however, was associated with assorted marriage by BMI and increased significantly by marriage years before and when the obesity prevalence began its second increase in Denmark. Future studies should investigate the potential role of upper BMI assortment on offspring susceptibility toward a high BMI, as well as the potential contribution to the general obesity epidemic.

## Author contributions

Teresa A. Ajslev and Thorkild I. A. Sørensen conceived the study. Teresa A. Ajslev, Lars Ängquist, Jennifer L. Baker, Michael Gamborg, and Thorkild I. A. Sørensen developed the study design. Teresa A. Ajslev and Lars Ängquist conducted the statistical analyses and wrote the first draft of the paper. All authors contributed to the critical review of the findings, the interpretations, as well as the preparation, and approval of the final manuscript.

### Conflict of interest statement

Dr. David B. Allison has, anticipates, or has had financial interests with the Frontiers Foundation; Vivus, Inc.; Kraft Foods; University of Wisconsin; University of Arizona; Paul, Weiss, Wharton and Garrison LLP; and Sage Publications. All other authors declare that they have no conflicts of interest.

## Footnotes

^1^Standardized BMI *z*-scores = BMI *z*-score minus gender-interval specific mean BMI *z*-score, divided by gender-interval specific standard deviation (SD).
